# Aberrant hippocampal gamma oscillations in a mouse model of fragile X syndrome: insights from in vitro slice models

**DOI:** 10.1186/s13229-025-00687-9

**Published:** 2025-11-03

**Authors:** Evangelia Pollali, Daniel Frías Donaire, Miguel Del Ángel, Yunus Emre Demiray, Sara Enrile Lacalle, Jan-Oliver Hollnagel, Anil Annamneedi, Gürsel Çalışkan

**Affiliations:** 1 Department of Genetics and Molecular Neurobiology, Institute of Biology, Otto-von-Guericke University, 39120 Magdeburg, Germany; 2 Research Group “Synapto-Oscillopathies”, Institute of Biology, Otto-von- Guericke-University, Leipziger Str. 44, Haus 91D, 39120 Magdeburg, Germany; 3 Institute of Physiology and Pathophysiology, University of Heidelberg, Im Neuenheimer Feld 326, 69120 Heidelberg, Germany; 4 Center for Behavioral Brain Sciences, Magdeburg, Germany; 5 School of Arts and Sciences, Sai University, OMR, Paiyanur, Tamil Nadu 603104 India; 6 Present Address: Institute of Physiology and Pathophysiology, Medical Faculty, Heidelberg University, 69120 Heidelberg, Germany

**Keywords:** Fragile x syndrome, *fmr1*, Knock-out, Hippocampus, Gamma oscillations, Sharp wave-ripples, Muscarinic receptor, Metabotropic glutamate receptor, Kainate receptor

## Abstract

**Background:**

Fragile X syndrome (FXS) is the most common inherited intellectual disability, caused by the loss of fragile X mental retardation protein (FMRP), which regulates neuronal signaling and plasticity. FXS patients and *Fmr1* knockout (KO) mice exhibit sensory hypersensitivity, hyperarousal, and hippocampus-dependent learning deficits. Dysregulated metabotropic glutamate receptor (mGluR) and muscarinic acetylcholine receptor (mAChR) signaling, along with reduced kainate receptor (KAR) function, have been implicated in FXS pathophysiology. Activation of these signaling pathways induce gamma-frequency network oscillations hippocampal slices in vitro. However, their specific contribution to aberrant gamma oscillations in FXS remains unclear.

**Methods:**

We recorded local field potential (LFP) gamma oscillations in vitro in hippocampal CA3 from wild-type (WT) and *Fmr1* KO mice. Oscillations were induced pharmacologically using carbachol (CCh), the group I mGluR agonist dihydroxyphenylglycine (DHPG), or kainate (KA). In addition, we quantified synaptic protein expression of mAChR M1, mGluR1, mGluR5, GluK1, and GluK2-receptors involved in gamma oscillation generation under these conditions.

**Results:**

*Fmr1* KO slices exhibited increased integrated gamma power (20–80 Hz) in response to DHPG and CCh, suggesting higher network synchronization through mGluR and mAChR pathways. In contrast, KA-induced oscillations showed reduced synchrony and gamma peak power, indicating disrupted network coordination. Aberrant spiking activity during both CCh- and KA-induced oscillations further supports impaired temporal coordination in *Fmr1* KO mice. These physiological changes were only partially reflected by altered expression of the corresponding receptor proteins.

**Limitations:**

In the current study, we found aberrant gamma oscillation power in in vitro hippocampal slices of *Fmr1* KO mice. It remains to be determined whether these oscillatory changes extend to pharmacologically induced gamma oscillations in cortical slice preparations in vitro.

**Conclusions:**

Our findings demonstrate that hippocampal gamma oscillations are differentially affected by distinct neuromodulatory pathways in *Fmr1* KO mice. Enhanced responsiveness to cholinergic and mGluR activation and reduced coherence of KA-induced rhythms suggest that multiple dysregulated mechanisms contribute to gamma oscillopathies in FXS.

**Supplementary information:**

The online version contains supplementary material available at 10.1186/s13229-025-00687-9.

## Background

Expansion of non-coding trinucleotide CGG above the normal range ( > 200 CGG repeats) causes the full mutation and loss of fragile X mental retardation protein (FMRP) leading to Fragile X syndrome (FXS), the most common genetically inherited form of intellectual disability and autism spectrum disorder [1, 2]. Lack of FMRP, a key translational regulator of the vast majority of synaptic proteins, leads to excessive protein synthesis, impaired synaptic function and dysregulation of protein-synthesis dependent plasticity [3, 4].

Accumulating evidence suggests that elevated muscarinic acetylcholine receptor (mAChR)-mediated signaling and metabotropic glutamate receptor5 (mGluR5)-mediated signaling are involved in the pathogenesis of FXS [5–7]. It is widely-accepted that loss of FMRP leads to excessive mGluR5-mediated protein synthesis and enhanced mGluR-mediated long-term depression (LTD) [5, 8, 9]. Importantly, both electrophysiological and behavioral phenotypes observed in the *Fmr1* KO mice can be corrected by genetic or pharmacological reduction of mGluR5 signaling [7, 10]. Similarly, *Fmr1* KO mice show increased expression of both type 1 (M1) and type 4 (M4) mAChR in the hippocampus [6]. Accordingly, hippocampal LTD induced by activation of mAChR is exaggerated [11] and antagonism of M1 can rescue some of the behavioral phenotypes observed in *Fmr1* KO mice [12]. On the other hand, ionotropic glutamate receptor function is also affected by lack of FMRP [13, 14]. Interestingly, a reduced kainate receptor (KAR)-mediated synaptic function has also been demonstrated in the cortex of the *Fmr1* KO mice [15].

Network oscillations are electrical brain activities reflecting neuronal synchronization at different spatial and temporal levels [16]. Neuronal network oscillations are crucial for maintaining most of the brain functions and can be used as proxies for neural mechanisms underlying behaviorally-relevant synaptic plasticity [17]. While the molecular and cellular mechanisms underlying FXS have been extensively studied in rodent models, systematic investigation of behaviorally-relevant neurophysiological oscillations as mesoscopic biomarkers for FXS has only recently accelerated [18–27]. Of note, the most striking and converging finding from studies using *Fmr1* KO mouse model is the gamma-range (30–80 Hz) oscillopathy observed in distinct cortical regions [18–23] and hippocampus [24–27]. Elevated gamma oscillations appear to be present during diverse behavioral stages including active exploration, rest and sleep [20, 24, 25]. Similar enhancement in the resting state gamma power and aberrant synchronization of gamma oscillations have been reported in electroencephalogram (EEG) studies in FXS patients [28–30]. These oscillatory changes have been associated with sensory and cognitive deficits in FXS [31]. Specifically, enhanced hippocampal gamma oscillations and their aberrant synchronization have been implicated in reduced cognitive flexibility in the *Fmr1* KO mice and autism spectrum disorders [26, 27, 32, 33]. Thus, aberrant gamma oscillations might indeed be a common mesoscopic biomarker for FXS and potentially for autism spectrum disorders. However, to date, it is still not clarified whether elevated mGluR signaling and/or mAChR signaling can indeed lead to an aberrant enhancement of gamma oscillations in the *Fmr1* KO mice.

In vitro oscillation models provide a robust tool for pharmacological studies aiming at elucidating phenotypic differences [34–36]. Accumulating evidence suggests that in vitro network oscillations engage similar cellular interactions as observed in vivo [37–39] and are sensitive to genetic and/or behavioral manipulations [34, 36, 40–44]. Specifically, the hippocampal CA3 subregion with its tightly interconnected recurrent network can generate gamma-range rhythmic local field potentials (LFP) in vitro [45, 46]. These LFP oscillations can be induced via activation of M1 [41, 47–49] or elevating glutamatergic input via activation of either mGluR1/5 [50, 51] or KAR [34, 47, 48, 52]. Activation of mAChRs, mGluR1/5, and KARs engages distinct excitatory and inhibitory components of the hippocampal CA3 circuitry to generate gamma oscillations. Specifically, mAChR and mGluR1/5 activation increases excitatory drive onto pyramidal neurons, promoting recurrent excitation, while KARs modulate both pyramidal cell excitability and interneuron recruitment [45, 50, 52]. Fast-spiking Parvalbumin-positive (PV+) interneurons provide phasic inhibition that synchronizes pyramidal neuron firing, producing coherent gamma rhythms [53, 54]. Thus, gamma oscillations emerge from the dynamic interplay between recurrent excitation among pyramidal cells and feedback inhibition from interneurons, with receptor-specific modulation shaping the amplitude and coherence of the rhythmic activity. In the absence of elevated cholinergic or glutamatergic input, hippocampal CA3 network generates spontaneous sharp wave-ripple (SW-R) activity in vitro that represents a distinct hippocampal network state [39, 55]. Thus, two distinct hippocampal LFP states observed in vivo can partially be mimicked using hippocampal slice preparations.

In the current study, we recorded in vitro hippocampal network oscillations in the *Fmr1* KO mice and analyzed spontaneous SW-Rs and pharmacologically induced gamma oscillations via activating mAChR, mGluR1/5 or KAR. Based on the elevated mAChR- and mGluR5-mediated signaling in the *Fmr1* KO mice, we postulated that direct activation of these signaling pathways in vitro would be sufficient to observe the aberrantly elevated gamma oscillations in *Fmr1* KO mice. Our findings provide promising evidence for direct involvement of elevated mAChR- and mGluR5-mediated signaling in the gamma-range oscillopathy observed in FXS.

## Methods

### Animals

We used male *Fmr1*-KO (–/y) mice and wild-type littermates (+/y) on an FVB background (JAX stock #003024) to ensure comparability with previous studies using this well-characterized fragile X line [56]. The FVB strain carries the *Pde6b* mutation causing early-onset retinal degeneration [57]. Our experiments were conducted in acute hippocampal slices from naïve 4–5-week-old mice without prior behavioral testing. At this age, retinal degeneration is already advanced, but visual input does not contribute to in vitro gamma oscillations generated in the isolated hippocampus recorded in the current study. Thus, the choice of this background does not confound our electrophysiological measurements and allows direct comparison to the established *Fmr1*-KO model maintained on the FVB strain. Naive mice were bred in our animal facility at the Institute of Biology, Otto-von-Guericke University Magdeburg (reverse 12 h light/dark cycle with lights switched on at 7 p.m., including a 30 min dawn phase; food and water ad libitum). Animals were euthanized by brief exposure to 5% isoflurane, and tissues collected postmortem. As no procedures were performed on live animals for scientific purposes, this protocol does not qualify as an animal experiment according to §7 Abs. 2 of the German Animal Welfare Act (TierSchG) and was therefore exempt from ethical approval.

### Electrophysiology

#### Slice preparation

Mice were anaesthetized with isoflurane and decapitated. The brain was rapidly removed and submerged in ice-cold artificial cerebrospinal fluid (aCSF) including (in mM) 129 NaCl, 21 NaHCO_3_, 3 KCl, 1.6 CaCl_2_, 1.8 MgCl, 1.25 NaH_2_PO4 and 10 glucose (carbogenated with 5% CO_2_, 95% O_2_, pH 7.4, osmolarity 300 mOsm). Horizontal slices containing ventral-to-mid hippocampus (400 µm) were prepared with a vibratome (Model 752; Campden Instruments LTD, Loughborough, UK) at an angle of about 12° in the fronto-occipital direction and placed in an interface chamber at 32 °C (aCSF flow rate: ~2 ml/min). Slices were allowed to recover for at least 1 h before starting the recordings.

### Local field potentials

LFP network oscillation recordings were obtained from stratum pyramidale (SP) of CA3 area. Borosilicate glass electrodes of ~ 1 MΩ resistance, filled with aCSF, were placed at a depth of ~ 80 µm and spontaneous SW-R were recorded. Gamma oscillations were induced by increasing the temperature to 35 °C and bath-application of drugs at least for 45 min. Temperature closer to physiological range increases the likelihood of observing peak frequencies above 30 Hz within the gamma band, which tend to shift to lower values at reduced temperatures [58, 59]. For each slice, recordings used for analysis were obtained over a 5 min period between 45 and 65 min post-drug application, when oscillations typically reach a plateau [59]. Recordings were terminated immediately afterward. To ensure comparability and reduce systematic bias, we recorded two slices simultaneously (one *Fmr1* KO and one WT) within the same interface chamber, and restricted recordings to 2–4 h post-preparation to maintain slice viability. The experimenter was blinded to the genotype. Importantly, slices from individual animals were used to record only one type of pharmacologically induced gamma oscillation (CCh, DHPG, or KA). This approach ensured that timing and slice health were comparable across genotypes for each experimental condition.

To obtain evoked field excitatory postsynaptic potential (fEPSP) responses from CA(Cornu Ammonis)3-to-CA1 synapse, the recording electrode was placed at the proximal apical dendrites of area CA1 and the bipolar stimulation electrode (exposed tips: ~20 µm; tip separations of ~ 75 µm; electrode resistance in aCSF: ~0.1 MΩ) was placed at the Schaffer collaterals (SC) at the proximal CA1 close to the CA2 subregion. After placing the electrodes, responses were recorded for 10-to-20 min until they were stabilized (inter-stimulus interval of 30 sec and stimulation duration of 100 µs). Then, an input-output (I-O) curve was recorded using intensities ranging from 10 to 50 µA. Slices were excluded if they exhibited fEPSP amplitude below 1 mV at 50 µA stimulation strength or showed polysynaptic responses that interfered with reliable measurement of the SC-evoked fEPSP slope. This was followed by a paired-pulse (PP) recording protocol with intervals ranging from 10 to 500 ms. These recordings were performed without pharmacological blockade of GABAergic transmission, thus reflecting mixed excitatory and inhibitory synaptic responses [60]. However, the initial slope of the fEPSP predominantly represents glutamatergic excitatory transmission mediated mainly by AMPA receptors and was therefore used as a measure of excitatory synaptic function (see below, section “Data Analysis”).

The signals were amplified by a commercial (EXT-02F-Extracellular Amplifier; npi Electronic GmbH, Tamm, Germany) or a custom-made amplifier and low-pass filtered at 3 kHz. The data were sampled at a rate of 5 kHz using a data acquisition unit (CED-Mikro1401; Cambridge Electronic Design, Cambridge, UK). Signals were recorded using Spike 2.8 software (Cambridge Electronic Design) and stored on a computer hard disc and analyzed off-line.

### Drug application

Drugs were applied via continuous perfusion with a flow rate of ~ 2 ml/min. AChR agonist Carbachol (CCh; 5 µM) (TOCRIS, Bristol, UK), mGluR1/5 agonist Dihydroxyphenylglycine (DHPG; 10 µM) (TOCRIS) and ionotropic KAR agonist Kainic acid (KA; 60 nM) (TOCRIS) were added to aCSF freshly prior to induction of gamma oscillations, using stock aliquots stored in −20 °C. CCh stocks were aliquoted in dimethyl sulfoxide (DMSO; Final Concentration: 0.01%) (Merck KGaA, Darmstadt, Germany) whereas DHPG and KA were aliquoted in distilled water.

### Data analysis

#### Gamma oscillations

**Fig. 1:**
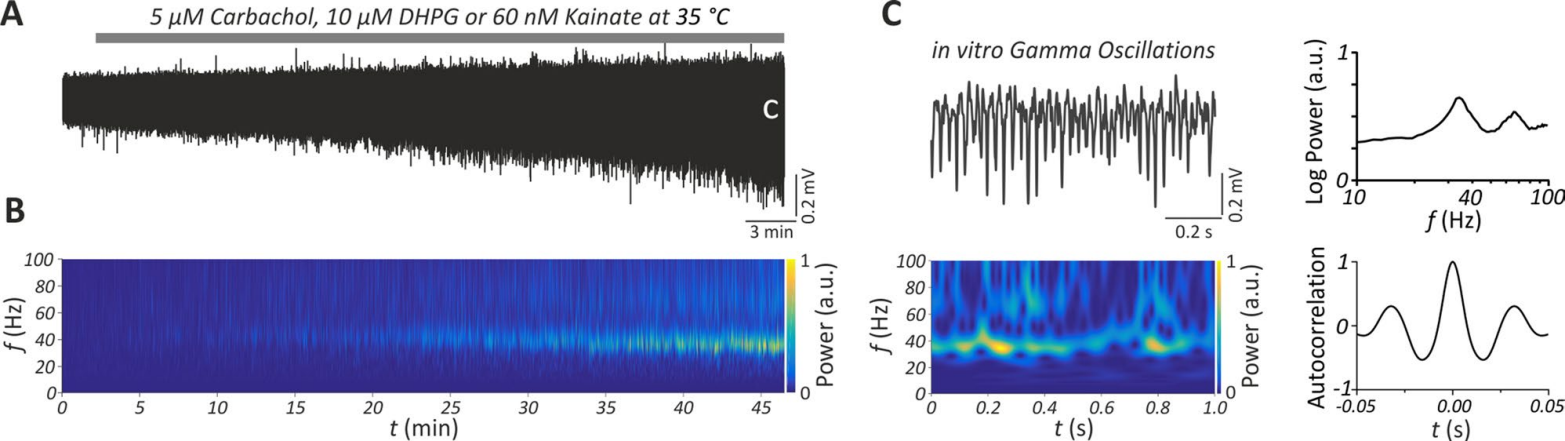
Gamma oscillations in the hippocampal CA3 in vitro. (**A**) sample trace of gamma oscillations induced via bath-application of either carbachol (CCh, 5 µM), DHPG (10 µM) or kainate (KA, 60 nM) for 45–60 min. Local field potential (LFP) recordings were performed from the pyramidal layer of CA3 subregion in horizontal brain slices including transverse-like sections of the ventral-to-mid hippocampus. “C” indicates the gamma oscillation trace shown in panel **C**. (**B**) Corresponding wavelet transformation of the LFP trace in panel a showing power of frequency domains (f) over time (t). Heat-scale colors encode for power in arbitrary units (a.U.). Note the emergence of gamma oscillations at 30–40 hz after 15 min of drug application and its stabilization after ~45 min. (**C**) Sample LFP gamma trace (left, up) and corresponding wavelet transformation (left, down) ~45 min after drug perfusion. Note the prominent gamma oscillations at ~ 40 hz. Power spectra (right, up) or auto-correlograms (right, down) were generated from artefact-free two min gamma oscillation segments at least 45 min after perfusion of CCh, DHPG or KA. Note that the *f* is shown in logarithmic scale

Pharmacologically induced gamma oscillations were recorded from pyramidal layer of CA3 region of ventral-to-mid hippocampus (Fig. 1). Two minutes artefact-free data were extracted and analyzed using Fast Fourier Transformation with a frequency resolution of 1.221 Hz with Spike 2.8 software (Cambridge Electronic Design) and custom-made MATLAB scripts (MathWorks, Natick, MA, USA). Peak frequency, integrated power (20–80 Hz) and half band width (HBW) were calculated from the power spectra of two-minute artifact free data. The slices without epileptiform discharges that show gamma oscillations with peak power greater than 10 µV^2^ and peak frequency greater than 20 Hz were included in the analysis. HBW was calculated as the frequency range at the half-maximum of the peak power. Autocorrelation was calculated as the 2^nd^ positive peak of two-minute auto-correlogram and time constant (Tau) was calculated as the decaying exponential fit to the peaks of the autocorrelation. The number of slices included for analysis, after applying the inclusion criteria, was similar between genotypes: for CCh, 24/32 (75%) in WT and 35/49 (71%) in *Fmr1* KO; for DHPG, 22/32 (69%) in WT and 26/34 (76%) in *Fmr1* KO; and for KA, 31/49 (63%) in WT and 30/43 (69%) in *Fmr1* KO.

### Multi-unit activity

Multi-unit activity during gamma oscillations was analyzed using filtering parameters similar to those described in previous studies [61]. In brief, gamma oscillations were band-pass filtered at 15–45 Hz (FFT filter) and further processed by a Hilbert transformation yielding the phase representation of gamma cycles. Unit activity was detected by extracting the high frequency component of the LFP signal (FFT filter: 500–3000 Hz). The band-pass filtered signal was then denoised by application of the wavelet denoising function implemented in MATLAB’s Wavelet Toolbox. For initial collection of units, only those with amplitudes bigger than 0.0125 mV were considered. Further evaluation was done with units representing the upper quartile of unit amplitudes. Unit timings were then related to the phase of the corresponding gamma cycle. For comparison of units and respective gamma cycles, gamma cycles and units’ spike timings were resampled. For each recording, the obtained gamma cycles were averaged separately. The polar distribution of units was normalized to represent the probability of unit firing within a gamma cycle. For statistical evaluation, the unit firing probability curves and their activation curves (i.e. cumulative firing probability) were plotted in two dimensions. Individual unit spikes comprising the multi-unit activity were further analyzed regarding their height, the width at half the height, the ratio of height-to-width, and the overall number of detected unit spikes within the two-minute windows. Of note, we compared multi-unit activity only in those experiments in which the gamma oscillations were clearly recorded in the pyramidal cell layer and thus providing representative activation curves. Note that in addition to Tau and the 2^nd^ positive peak of the autocorrelogram, the unit firing probability and activation curve shape also serve as valid proxies for synchrony.

### Sharp wave-ripples

Extracellular local field potential recordings in the CA3 subregion of the ventral-to-mid hippocampus exhibit spontaneous sharp wave-ripple (SW-R) activity in vitro [40, 55]. Two minutes artefact-free data were extracted and custom-made MATLAB scripts (MathWorks, Natick, MA, USA) were used to analyze the sharp wave (SW) incidence, SW area under curve, the ripple frequency and ripple amplitude. Specifically, SW were identified by low-pass filtering the raw data at 45 Hz (FFT filter, cut frequency: 45 Hz). Only events greater than 3 times the standard deviation (SD) of the low-pass-filtered signal and with a minimum distance of 100 ms between two consecutive SW were considered for analysis. SW area was calculated by using as start and end of a SW event, the points crossing the mean of the data. In order to isolate the ripple component, time windows of 125 ms centered to the maximum of SW event were kept and band-pass filtered at 120–300 Hz (FFT filter). Only ripples with amplitude greater than 3 times the SD of the band-pass-filtered signal were considered. Data with length of 15 ms before and 10 ms after the maximum of SW event were stored and ripple amplitude was calculated using triple-point-minimax-determination. Ripples were included in the analysis, when the difference between the falling and rising component of a ripple was lower than 75%. Ripple frequencies were calculated using the duration between the through of consecutive ripples within each SW-R complex.

### Evoked synaptic potentials

MATLAB-based analysis tools were used for the analysis of fEPSPs (MathWorks, Natick, MA). For calculation of fEPSP slopes, the slope (V/s) between the 20 and 80% of the fEPSP amplitudes were measured. Average baseline transmission rate per slice was calculated by dividing each fEPSP slope value with the corresponding FV value followed by averaging these values leading to one transmission rate (ms^−1^) value per slice. Paired-pulse responses were analyzed by dividing the slope of the second fEPSP to the first one.

### Protein expression

#### Preparation of enriched synaptosome fraction

The ventral to mid-portion of the hippocampus, corresponding to the region used for electrophysiological recordings, was dissected to obtain enriched synaptosome fraction. The tissue was collected in an Eppendorf tube and mechanically homogenized on ice using a disposable pestle in 200 μl of Syn-PER extraction reagent (ThermoFisher, 87,793), supplemented with Pierce protease inhibitor (ThermoFisher, A32963). The homogenate was centrifuged at 1200 ×g for 10 minutes at 4 °C. The resulting supernatant was transferred to a new tube and centrifuged again at 15,000 ×g for 20 minutes at 4 °C. The final supernatant, containing the cytosolic fraction, was collected in a fresh Eppendorf tube. The pellet, containing the enriched synaptosome fraction, was resuspended in 150 μl of Syn-PER extraction reagent. Protein concentration was determined using a modified Lowry assay (BIO-RAD, 5,000,111) according to the manufacturer’s instructions.

### Western blot analysis

Samples enriched in cytosolic and synaptosome fractions were prepared for Western blot analysis by boiling in sample buffer (40% glycerol, 240 mM Tris-HCl pH 6.8, 8% SDS, 0.04% bromophenol blue, 5% β-mercaptoethanol) for 5 minutes. Equal amounts of protein (20 μg per sample) were loaded onto SDS-PAGE gels for electrophoresis, and then electrotransferred to Immobilon FL-PVDF membranes (Millipore, IPFL00010). The membranes were blocked for 1 hour in Intercept blocking buffer (LI-COR, 927–60001), followed by overnight incubation at 4 °C with primary antibodies diluted in the same buffer (Table 1). Protein detection was carried out using either near-infrared-labelled or HRP-conjugated secondary antibodies, depending on the assay. Near-infrared signals were visualized with an Odyssey scanner, while chemiluminescent signals were detected using the Odyssey FC imaging system (LI-COR). Signal quantification and normalization were performed using Image Studio Lite software (LI-COR). For mGluR5, which typically appears as a diffuse smear in membrane-rich synaptosome fractions, the entire signal corresponding to the full-length, heavily glycosylated receptor was quantified [62, 63], consistent with previous reports using the same antibody [64, 65].

**Table 1:** Antibody list

Target	Host	Brand	Cat. No.	Concentration
GluK1	Mouse	Synaptic Systems	114011	1:1000
GluK2	Rabbit	Synaptic Systems	180003	1:1000
GABA _A_R-α1	Guinea Pig	Synaptic Systems	224205	1:1000
GABA _A_R-α2	Rabbit	Synaptic Systems	224103	1:1000
M1	Goat	Abcam	Ab77098	1:1000
mGLUR1-α	Rabbit	Synaptic Systems	191002	1:1000
mGLUR5	Rabbit	Cell Signaling	55920	1:1000
PSD95	Mouse	Abcam	Ab13552	1:1000
Tubulin	Rabbit	LI-COR	926–42211	1:1000
Tubulin	Mouse	Sigma-Aldrich	T6199	1:10000
Mouse IgG 680	Donkey	LI-COR	926–68072	1:50000
Rabbit IgG 680	Donkey	LI-COR	926–68073	1:50000
Mouse IgG 800	Donkey	LI-COR	926–32212	1:50000
Rabbit IgG 800	Donkey	LI-COR	926–32213	1:50000
Goat IgG 800	Donkey	LI-COR	926–32224	1:50000

### Statistical analysis

The number of animals (N) and the number of slices (n) used are indicated in the figure legends. GraphPad Prism for Windows (GraphPad Software, CA, USA) was used for statistical comparison. Consistently and blindly for all data Shapiro–Wilk test was used to determine whether the data were normally distributed. For oscillation and protein expression data, the genotype differences were determined either by Student’s two-tailed t-test for normally distributed data and Mann-Whitney U test was used otherwise. Log transformation was used for the statistical comparison of gamma power due to the variable nature of gamma oscillation power in vitro. To provide information about differences in the multi-unit activity relative to the gamma cycle multiple t-test comparison was applied without assuming a similar standard deviation. The statistical comparisons of evoked electrophysiological responses across different stimulation strengths (IO curves) and intervals (paired-pulse responses) were done by two-way repeated measures ANOVA followed by post-hoc comparison using Holm-Šídák multiple comparison test with Greenhouse-Geisser correction. Cohen’s d was used to compute the effect size and 95% confidence interval is reported. Data are reported as mean ± standard error of the mean (SEM) or presented as Tukey-style boxplots, where the central line indicates the median, the box spans the interquartile range (IQR; 25th–75th percentile), and whiskers extend to 1.5× IQR. Points outside the whiskers represent individual outliers. Probability value at least *p* < 0.05 was considered significant.

## Results

### Cholinergic gamma oscillations are enhanced in the CA3 of *Fmr1* KO mice

**Fig. 2:**
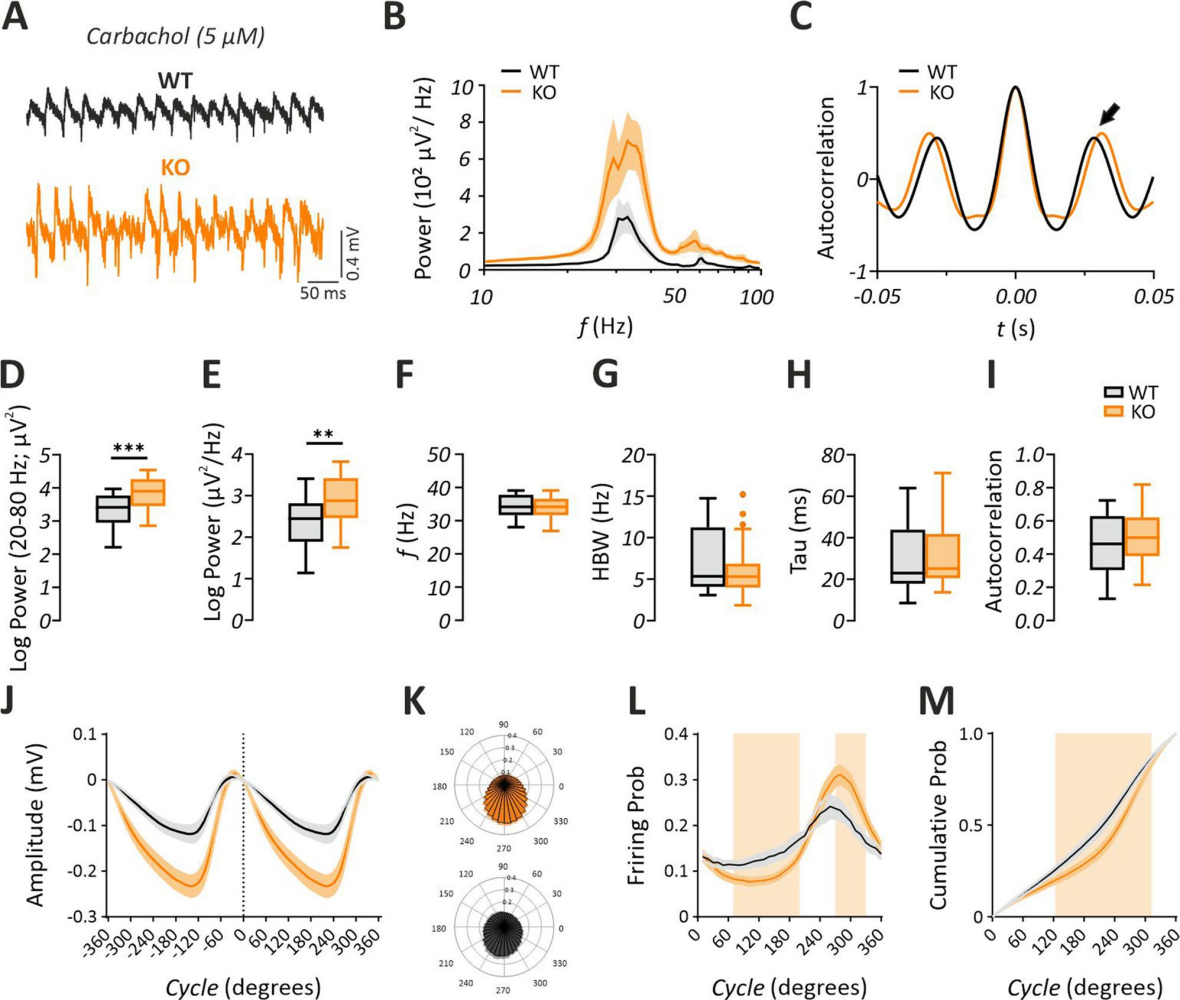
Carbachol-induced cholinergic gamma oscillations are augmented in the hippocampal CA3 subregion of *Fmr1* KO mice in vitro. (**A**) sample trace of gamma oscillations induced via bath-application of carbachol (CCh, 5 µM). (**B**) Average power spectra of gamma oscillations showing an increase in the power of gamma-range (20–80 hz) LFP oscillations without any shift in the gamma peak frequency (*f*). Note that the *f* is shown in logarithmic scale. (**C**) Representative autocorrelogram showing no change between genotypes. The 2nd positive peak value (indicated by the arrow) was measured for statistical comparison. Summary plots highlighting (**D**) a significant increase in the gamma power (20–80 Hz) (WT: *n* = 24/*N* = 8, KO: *n* = 35/*N* = 11) and in (**E**) peak power (WT: *n* = 24/*N* = 8, KO: *n* = 35/*N* = 11), no significant alterations in (**F**) the gamma peak f (WT: *n* = 24/*N* = 8, KO: *n* = 35/*N* = 11), (**G**) half band width (HBW) (WT: *n* = 18/*N* = 8, KO: *n* = 30/*N* = 11), (**H**) time constant (tau) of the decaying exponential fit to the peaks of the autocorrelation (WT: *n* = 18/*N* = 8, KO: *n* = 30/*N* = 11) and (**I**) 2nd positive peak value of the autocorrelogram (WT: *n* = 18/*N* = 8, KO: *n* = 30/*N* = 11). Evaluation of multi-unit activity in datasets (WT: *n* = 12/*N* = 6, KO: *n* = 25/*N* = 8) is shown in **J-M**. (**I**) Average gamma cycle. Note that the cycle’s amplitude is plotted against degrees. (**K**) Polar plots illustrating the probability of unit activity in *Fmr1* KO (top) and WT (bottom) mice relative to the cycle shown in **J**. (**L**) Unit firing probability reveals different activation regimes. Units in WT are significantly more active in the initial phase, whereas units in *Fmr1* KO mice have a significantly higher firing probability in the late phase. (**M**) Unit activation curves reveal lower phase specificity in WT mice compared to *Fmr1* KO mice during gamma cycles, indicating that units in WT fire more evenly across the cycle. Statistical comparison was performed using Student´s two-tailed t-test in **D**, **E**, **I** and mann-whitney U test in **F-H**. In **L** and **M** statistical comparison was done with multiple t-test comparisons. Colored patches indicate ranges in which datasets differed significantly. In **B** and **J-M** data are presented as mean ± SEM. In **D-I** data are presented as Tukey-style boxplots, where the central line indicates the median, the box spans the interquartile range (IQR; 25th–75th percentile), and whiskers extend to 1.5× IQR. Points outside the whiskers represent individual outliers. ***p < 0.001, **p < 0.01

Converging evidence indicates that mAChR signaling is elevated in the *Fmr1* KO mice [6, 11, 12]. Furthermore, induction of cholinergic-type gamma oscillations depends on activation of mAChR [45, 49]. Thus, we first characterized cholinergic gamma oscillations in the hippocampal CA3 subregion of *Fmr1* KO and WT mice via bath-application of 5 µM CCh. Gamma oscillations emerged 10–15 min after application of CCh and stabilized after ~45 min (Fig. 2A, Table 2). Strikingly, we found a profoundly increased gamma power (20–80 Hz) in the *Fmr1* KO compared to the WT mice (Fig. 2B, D, J; Student’s two-tailed t-test; T [57] = 4.055, *p* = 0.0002 with a large effect size d = −1.07, 95% CI [−1.63, −0.52]). Similarly, peak power was increased in *Fmr1* KO compared to WT (Fig. 2B, E, J; Student’s two-tailed t-test; T [57] = 3.410, *p* = 0.0012 with a large effect size d = −0.90, 95% CI [−1.44, −0.35]). However, analysis of the gamma peak frequency (Fig. 2F; Mann-Whitney U test, *p* = 0.9418), the half band width (Fig. 2G; Mann-Whitney U test, *p* = 0.7477), the decay constant gamma autocorrelation fit (Tau) (Fig. 2H; Mann-Whitney U test, *p* = 0.5094) and the autocorrelation (2^nd^ peak value) of local gamma oscillations (Fig. 2C, I; Student’s two-tailed t-test; T [46] = 0.9627, *p* = 0.3407) revealed no genotype differences. Of note, we observed significant differences in the unit activity which corresponds to an overall neuronal activity that reflect action potentials generated by both pyramidal neurons and interneurons (Fig. 2J–K). While the probability of unit firing was slightly higher at the beginning of each gamma cycle in WT mice, cells were more active in the late phase of gamma cycles in *Fmr1* KO mice (Fig. 2L–M; multiple t-test comparisons; *p* < 0.05, indicated by colored patches). Spike waveform analysis revealed a significant increase in the amplitude of individual unit spikes contributing to the multi-unit activity in *Fmr1* KO mice under CCh (Supplementary Fig. 1). Together, these data suggest that cholinergic gamma oscillations in the hippocampus of *Fmr1* KO mice are substantially augmented and may underlie the aberrantly enhanced gamma oscillations observed in *Fmr1* KO mice in vivo.

**Table 2:** Gamma parameters (mean ± SEM) after CCh, DHPG and KA, with corresponding p-values for direct comparisons

		log power 20–80 Hz			log power		
		mean	SEM	p value	mean	SEM	p value
CCh	WT	3.329	0.09448	**0.0002**	2.4	0.1164	**0.0012**
	KO	3.825	0.07796		2.903	0.09264	
DHPG	WT	3.082	0.09997	**0.0345**	1.829	0.1365	0.0715
	KO	3.383	0.09476		2.165	0.1214	
KA	WT	3.394	0.1046	0.1537	2.226	0.1225	**0.0378**
	KO	3.193	0.09088		1.922	0.1014	
		**peak f**			**HBW**		
		mean	SEM	p value	mean	SEM	p value
CCh	WT	34.29	0.7064	0.9418	7.083	0.9697	0.7477
	KO	34.11	0.5546		6.168	0.5843	
DHPG	WT	44.44	1.396	0.4209	8.163	1.307	0.4876
	KO	45.82	1.028		7.902	0.7298	
KA	WT	34.03	0.9033	**0.0491**	10.72	1.139	0.0598
	KO	36.82	1.064		12.01	0.8569	
		**tau**			**autocorrelation**		
		mean	SEM	p value	mean	SEM	p value
CCh	WT	30.39	4.07	0.5094	0.4546	0.04817	0.3407
	KO	31.57	2.654		0.5049	0.02843	
DHPG	WT	17.51	2.681	0.7788	0.4076	0.05766	0.5267
	KO	19.61	3.027		0.3629	0.04226	
KA	WT	19.71	1.506	**0.0036**	0.3031	0.02697	0.0508
	KO	13.78	1.065		0.2275	0.02528	

### DHPG-induced gamma oscillations are enhanced in the CA3 of *Fmr1* KO mice

**Fig. 3:**
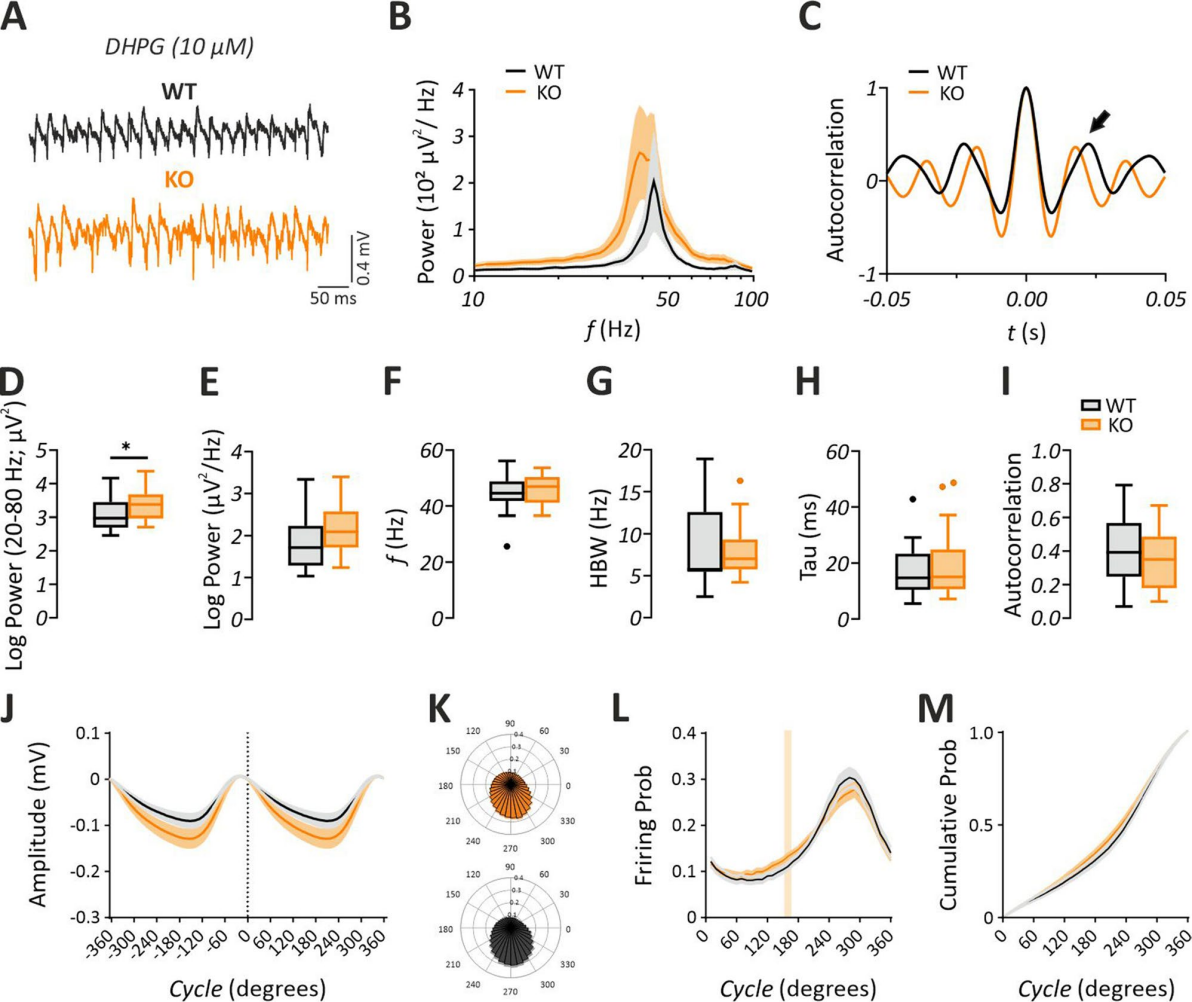
DHPG-induced gamma oscillations are augmented in the hippocampal CA3 subregion of *Fmr1* KO mice in vitro. (**A**) sample trace of gamma oscillations induced via bath-application of dihydroxyphenylglycine (DHPG, 10 µM). (**B**) Average power spectra of gamma oscillations showing an increase in the power of gamma-range oscillations without any shift in the gamma peak oscillation frequency (*f*). Note that the *f* is shown in logarithmic scale. (**C**) Representative autocorrelogram showing no change between genotypes. 2nd positive peak value (indicated by the arrow) was measured for statistical comparison. Summary plots illustrating a significant increase in (**D**) the gamma power (20–80 hz) (WT: *n* = 22/*N* = 9, KO: *n* = 26/*N* = 8) and no alteration (**E**) in the gamma peak power (WT: *n* = 22/*N* = 9, KO: *n* = 26/*N* = 8), (**F**) peak *f* (WT: *n* = 22/*N* = 9, KO: *n* = 26/*N* = 8), (**G**) half band width (HBW) (WT: *n* = 14/*N* = 7, KO: *n* = 18/*N* = 7), (**H**) time constant (Tau) of the decaying exponential fit to the peaks of the autocorrelation (WT: *n* = 14/*N* = 7, KO: *n* = 18/*N* = 7) and (**I**) 2nd peak value of the autocorrelogram (WT: *n* = 14/*N* = 7, KO: *n* = 18/*N* = 7). Evaluation of multi-unit activity in datasets (WT: *n* = 20/*N* = 8, KO: *n* = 24/*N* = 8) is shown in **J-M**. (**J**) Average gamma cycle. Note that the cycle’s amplitude is plotted against degrees. (**K**) Polar plots illustrating the probability of unit activity in *Fmr1* KO (top) and WT (bottom) mice relative to the cycle shown in **J**. (**L**) Unit firing probability showing similar activation regimes. (**M**) Unit activation curves with similar recruitment of units in WT vs. *Fmr1* KO mice. Statistical comparison was performed using Student´s two-tailed t-test in **D-F**, **I** and mann-whitney U test in **G** and **H**. In **L** and **M** statistical comparison was done with multiple t-test comparisons. Colored line in **K** indicates significant difference. In **B** and **J-M** data are presented as mean ± SEM. In **D-I** data are presented as Tukey-style boxplots, where the central line indicates the median, the box spans the interquartile range (IQR; 25th–75th percentile), and whiskers extend to 1.5× IQR. Points outside the whiskers represent individual outliers. *p < 0.05

In addition to mAChR signaling, increased mGluR signaling is also evident in the *Fmr1* KO mice [7, 10]. Activation of mGluR1/5 signaling in the hippocampus in vitro also induces gamma-range LFP oscillations [50, 51]. Thus, we characterized the gamma network activity induced by activation of mGluR1/5 via DHPG (Fig. 3A, Table 2). We found that the power of DHPG-induced gamma oscillations was significantly increased in the *Fmr1* KO compared to WT (Fig. 3B, D, J; Student’s two-tailed t-test; T [46] = 2.179, *p* = 0.0345, with a medium effect size d = −0.63, 95% CI [−1.21, −0.049]). As reported previously [50], DHPG-induced gamma oscillations showed an increased gamma peak frequency in comparison to the CCh-induced gamma oscillations (DHPG: 44.4 ± 1.5 Hz vs. CCh: 34 ± 0.2 Hz). However, no genotype differences were detected for gamma peak power (Fig. 3E; Student’s two-tailed t-test; T [46] = 1.845, *p* = 0.0715), peak frequency (Fig. 3F; Student’s two-tailed t-test; T [46] = 0.8122, *p* = 0.4209). Similarly, HBW (Fig. 3G; Mann-Whitney U test, *p* = 0.4876), Tau (Fig. 3H; Mann-Whitney U test, *p* = 0.7788) and gamma autocorrelation (Fig. 3C, I; Student’s two-tailed t-test; T [30] = 0.6405, *p* = 0.5267) were comparable between *Fmr1* KO and WT mice. Thus, in addition to the enhanced cholinergic signaling, mGluR signaling might also be directly involved in the enhancement of gamma oscillations in the *Fmr1* KO mice. Interestingly, the evaluation of the multi-unit activity during gamma cycles did not reveal a meaningful difference between WT and *Fmr1* KO mice (Fig. 3J–M; multiple t-test comparisons; *p* > 0.05). Similarly, analysis of basic spike waveform features (e.g., amplitude, width) revealed no significant differences between genotypes under DHPG (Supplementary Fig. 1).

### Synchronization of KA-induced gamma oscillations is reduced in the hippocampal CA3 of *Fmr1* KO mice

**Fig. 4:**
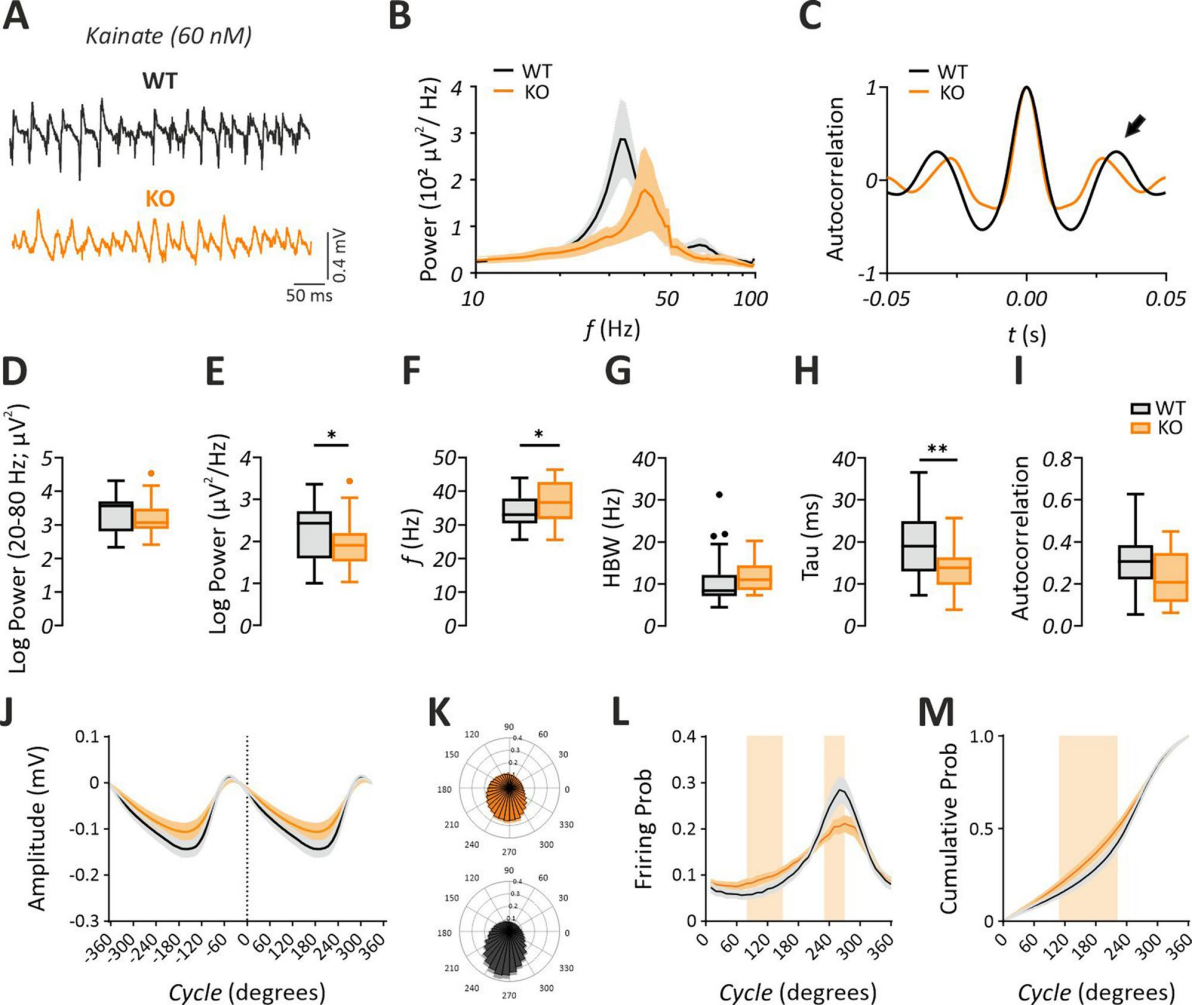
Kainate-induced gamma oscillations are reduced in power and synchrony in the hippocampal CA3 of *Fmr1* KO mice in vitro. (**A**) sample trace of gamma oscillations induced via bath-application of kainate (KA, 60 nM). Note the increased peak frequency and altered synchronicity in Fmr1 KO slices, reflected by a reduced time constant (tau) of the decaying exponential fit to the peaks of the autocorrelation, compared to WT. (**B**) Average power spectra of gamma oscillations showing a shift in the gamma peak oscillation frequency (*f*). (**C**) Corresponding autocorrelogram showing a slight reduction in the 2nd positive peak value (indicated by the arrow) in the *Fmr1* KO. Summary plots illustrating no changes (**D**) in power (20–80 hz) (WT: *n* = 31/*N* = 7, KO: *n* = 30/*N* = 7), but a significant decrease in (**E**) the peak power (WT: *n* = 31/*N* = 7, KO: *n* = 30/*N* = 7), increase in the (**F**) the gamma peak *f* (WT: *n* = 31/*N* = 7, KO: *n* = 30/*N* = 7) and a significant decrease in (**H**) Tau of the decaying exponential fit to the peaks of the autocorrelation (WT: *n* = 29/*N* = 7, KO: *n* = 23/*N* = 7), without strong alterations in), (**G**) half band width (HBW) (WT: *n* = 29/*N* = 7, KO: *n* = 23/*N* = 7) and (**I**) autocorrelation value (2nd peak value of the autocorrelogram) (WT: *n* = 29/*N* = 7, KO: *n* = 23/*N* = 7). Evaluation of multi-unit activity in datasets (WT: *n* = 27/*N* = 6, KO: *n* = 25/*N* = 6) is shown in **J-M**. (**J**) Average gamma cycle. Note that the cycle’s amplitude is plotted against degrees. (**K**) Polar plots illustrating the probability of unit activity in *Fmr1* KO (top) and WT (bottom) mice relative to the cycle shown in **J**. (**L**) Unit firing probability reveals different activation regimes. Units in WT are significantly more active in the late phase, whereas units in *Fmr1* KO mice have a significantly higher firing probability in the early phase. (**M**) Unit activation curves reveal more evenly distributed recruitment of units in *Fmr1* KO mice. Statistical comparison was performed using Student´s two-tailed t-test in **D**, **F**, **H**, **I** and mann-whitney U test in **E** and **G**. In **L** and **M** statistical comparison was done with multiple t-test comparisons. Colored patches indicate ranges in which datasets differed significantly. In **B** and **J-M** data are presented as mean ± SEM. In **D-I** data are presented as Tukey-style boxplots, where the central line indicates the median, the box spans the interquartile range (IQR; 25th–75th percentile), and whiskers extend to 1.5× IQR. Points outside the whiskers represent individual outliers. **p < 0.01, *p < 0.05

KAR activation has been a standard protocol for investigation of gamma oscillations in vitro [66]. In comparison to mAChR and mGluR signaling, a possible alteration in the ionotropic KAR signaling in the pathogenesis of FXS has been inadequately addressed. Thus, we aimed to characterize KA-induced gamma oscillations in the hippocampal CA3 of *Fmr1* KO mice (Fig. 4A, Table 2). In contrast to the increased gamma power after CCh or DHPG, we found no alteration in the power (20–80 Hz) of KA-induced gamma oscillations (Fig. 4B, D, J; Student’s two-tailed t-test; T [59] = 1.445, *p* = 0.1537), but interestingly observed a significant decrease in the peak power (Fig. 4B, E; Mann-Whitney U test; *p* = 0.0378, with a medium effect size d = 0.48, 95% CI [−0.02, 0.99]. Additionally, we found a significant increase in the peak frequency of gamma oscillations (Fig. 4B, F; Student’s two-tailed t-test; T [59] = 2.009, *p* = 0.0491, with medium effect size d = −0.51, 95% CI [−1.02, −0.002]). A tendency for an increased HBW (Fig. 4G; Mann-Whitney U test; *p* = 0.0598) was evident suggesting a possible change in the gamma synchronicity. Accordingly, analysis of time constant (Tau) of the decaying exponential fit to the peaks of the autocorrelation revealed a significant reduction (Fig. 4H; Student’s two-tailed t-test; T [50] = 3.056, *p* = 0.0036 with a large effect size d = 0.85, 95% CI [0.28, 1.42]) and a tendency for decreased autocorrelation (Fig. 4C, I; Student’s two-tailed t-test; T [50] = 2.001, *p* = 0.0508) in *Fmr1* KO mice compared to WT. At the multi-unit level and opposed to the findings described for CCh-induced gamma oscillations, we detected a more evenly distributed activation curve in the *Fmr1* KO mice (Fig. 4J–M; multiple t-test comparisons; *p* < 0.05, indicated by colored patches). Here, the probability of unit firing was slightly higher at the beginning of the gamma cycle in *Fmr1* KO mice, while it was lower towards the end of the gamma cycle (Fig. 4L; multiple t-test comparisons; *p* < 0.05, indicated by colored patches). However, basic spike waveform features were comparable between genotypes under KA (Supplementary Fig. 1). These data suggest a moderate reduction in the synchronicity and power of KA-induced gamma oscillations and aligns with a reduction KAR-mediated synaptic function reported in the cortex of *Fmr1* KO mice [15].

### Baseline gamma power is already enhanced in the hippocampal CA3 of Fmr1 KO mice

To determine whether gamma-band (20–80 Hz) oscillatory activity is already elevated at baseline in the hippocampal CA3 of *Fmr1* KO mice, we performed power spectrum analysis on slices exhibiting spontaneous activity (Supplementary Fig. 2A-B). Notably, the integrated 20–80 Hz power was significantly increased in *Fmr*1 KO slices compared to WT (Supplementary Fig. 2B; Student’s two-tailed t-test: T [67] = 2.475, *p* = 0.0151 with a medium effect size d = −0.50, 95% CI [−0.91, −0.09]). These results indicate that cholinergic and metabotropic glutamatergic signaling may already be more engaged under baseline conditions leading already a relatively mild but significant enhancement of gamma power in *Fmr1* KO slices.

### Spontaneous SW-R incidence is lower in the hippocampal CA3 of *Fmr1* KO mice

**Fig. 5:**
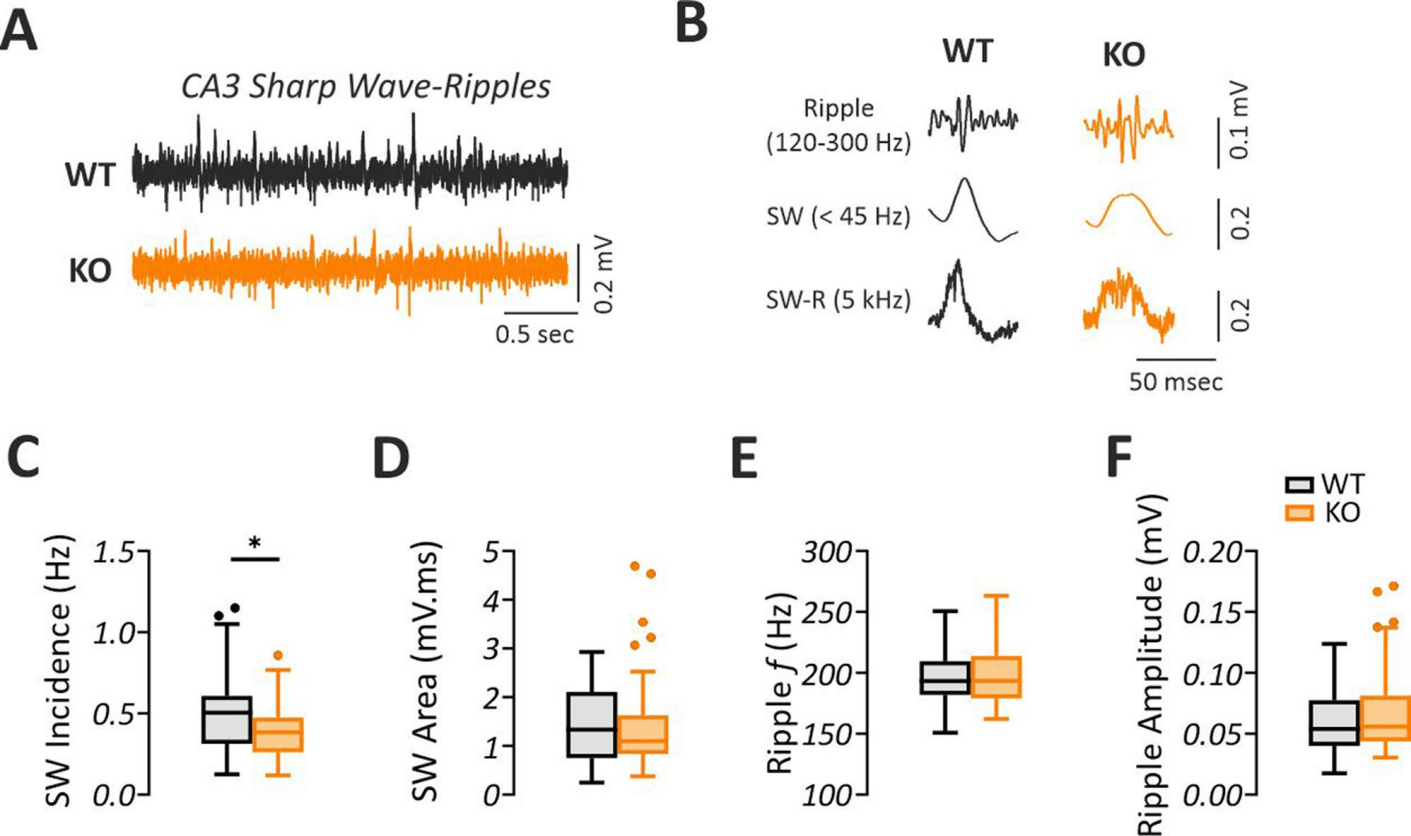
Lower SW-R rate in the hippocampal CA3 of *Fmr1* KO mice in vitro. (**A**) Sample traces of spontaneous sharp wave-ripples (SW-R) in the CA3 of WT and *Fmr1* KO mice, illustrating a lower number of SW-R events. (**B**) Filtered SW-R traces showing the ripple component (band-pass filtered signal (120–300 hz)) and the sharp wave component (low-pass filtered signal ( < 45 hz)). Summary graphs showing (**C**) decreased incidence in the *Fmr1* KO compared to the WT, with no changes in (**D**) SW area, (**E**) ripple frequency (*f*) and (**F**) ripple amplitude. Statistical comparison was performed using Student´s two-tailed t-test in **C** and mann-whitney U test in **D**, **E** and **F**. Data are presented as Tukey-style boxplots, where the central line indicates the median, the box spans the interquartile range (IQR; 25th–75th percentile), and whiskers extend to 1.5× IQR. Points outside the whiskers represent individual outliers. *p < 0.05. (WT: *n* = 43/*N* = 11, KO: *n* = 54/*N* = 14)

Under reduced subcortical neuromodulation, the same hippocampal circuits can generate SW-R activity [39]. Thus, we assessed different parameters of SW-R in the hippocampal CA3 subregion of *Fmr1* KO and WT mice (Fig. A-B). We found that SW-R incidence was decreased in *Fmr1* KO mice compared to WT (Fig. 5C; Mann-Whitney U test, *p* = 0.0473, with a medium effect size d = 0.48, 95% CI [0.08, 0.89]). However, we did not find any differences in other SW-R properties including SW area (Fig. 5D; Mann-Whitney U test, *p* = 0.4989), ripple frequency (Fig. 5E; Mann-Whitney U test, *p* = 0.7313) and ripple amplitude (Fig. 5F; Mann-Whitney U test, *p* = 0.6776). These data indicate that the occurrence of SW-R in the ventral-to-mid hippocampus in vitro is decreased in the *Fmr1* KO mice.

### Baseline synaptic transmission and short-term plasticity at the CA3–CA1 synapse are not altered in *Fmr1* KO mice

**Fig. 6:**
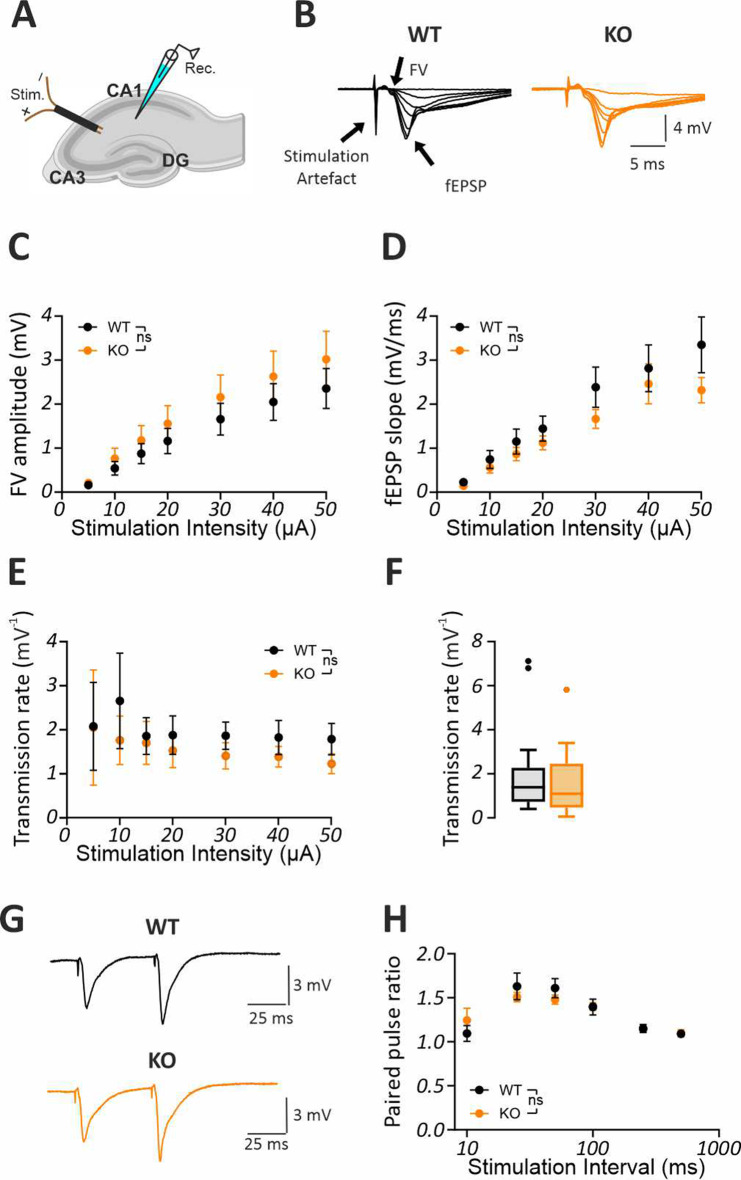
Unaltered baseline excitability and short-term plasticity in the schafer collateral (SC)-CA1 synapse of *Fmr1* KO mice. (**A**) sketch depicting the positioning of the stimulation and recording electrode to record evoked fEPSP responses at the SC-CA1 synapse of ventral-to-mid hippocampus. (**B**) Sample CA1 fEPSP traces showing comparable responses to increasing stimulation strengths in WT and *Fmr1* KO mice. Input-output curve (WT: *n* = 18/*N* = 12, KO: *n* = 15/*N* = 11) for (**C**) presynaptic FV and (**D**) postsynaptic fEPSP revealed no genotype effects. (**E**) Transmission rates for each stimulation strength and (**F**) transmission rate averaged per slice showed no statistical differences between the genotypes. (**G**) Sample paired-pulse response at 50 ms illustrating comparable facilitation in the WT and *Fmr1* KO mice. (**H**) Paired-pulse responses were comparable between the genotypes (WT: *n* = 19/*N* = 11, KO: *n* = 9/*N* = 11). Statistical comparison was performed with two-way repeated measures ANOVA followed by post-hoc comparison using holm-Šídák multiple comparison test with greenhouse–geisser correction in **C**, **D**, **E** and **H**. Mann-whitney U test was used in **F**. Data are presented as mean ± SEM. In F data are presented as Tukey-style boxplots, where the central line indicates the median, the box spans the interquartile range (IQR; 25th–75th percentile), and whiskers extend to 1.5× IQR. Points outside the whiskers represent individual outliers. ns = not significant

To elucidate if the identified alterations in hippocampal network oscillations are associated with changes in hippocampal synaptic transmission, we recorded evoked CA1 fEPSPs in response to Schaffer collateral (SC) stimulation (Fig. 6A, B). We found no evidence for changes in either presynaptic fiber volley (FV) (Fig. 6C; F [1, 31] = 0.6914, *p* = 0.412) or postsynaptic fEPSP responses (Fig. 6D; F [1, 31] = 1.170, *p* = 0.2877). Transmission rates (fEPSP slopes/FV amplitudes) were also not altered between the genotypes (Fig. 6E; F [1, 31] = 0.4335, *p* = 0.5151 and Fig. 6F; Mann-Whitney U test, *p* = 0.4638). Similarly, short-term plasticity assessed by analysing paired-pulse responses to different stimulation intervals revealed no genotype effects (Fig. 6G, H; F [1, 29] = 0.02868, *p* = 0.8667). These data indicate that hippocampal network oscillations are altered without any impact on baseline synaptic transmission and short-term plasticity in the *Fmr1* KO mice.

### Expression of receptors linked to in vitro hippocampal gamma oscillations is only partially altered in *Fmr1* KO mice

**Fig. 7:**
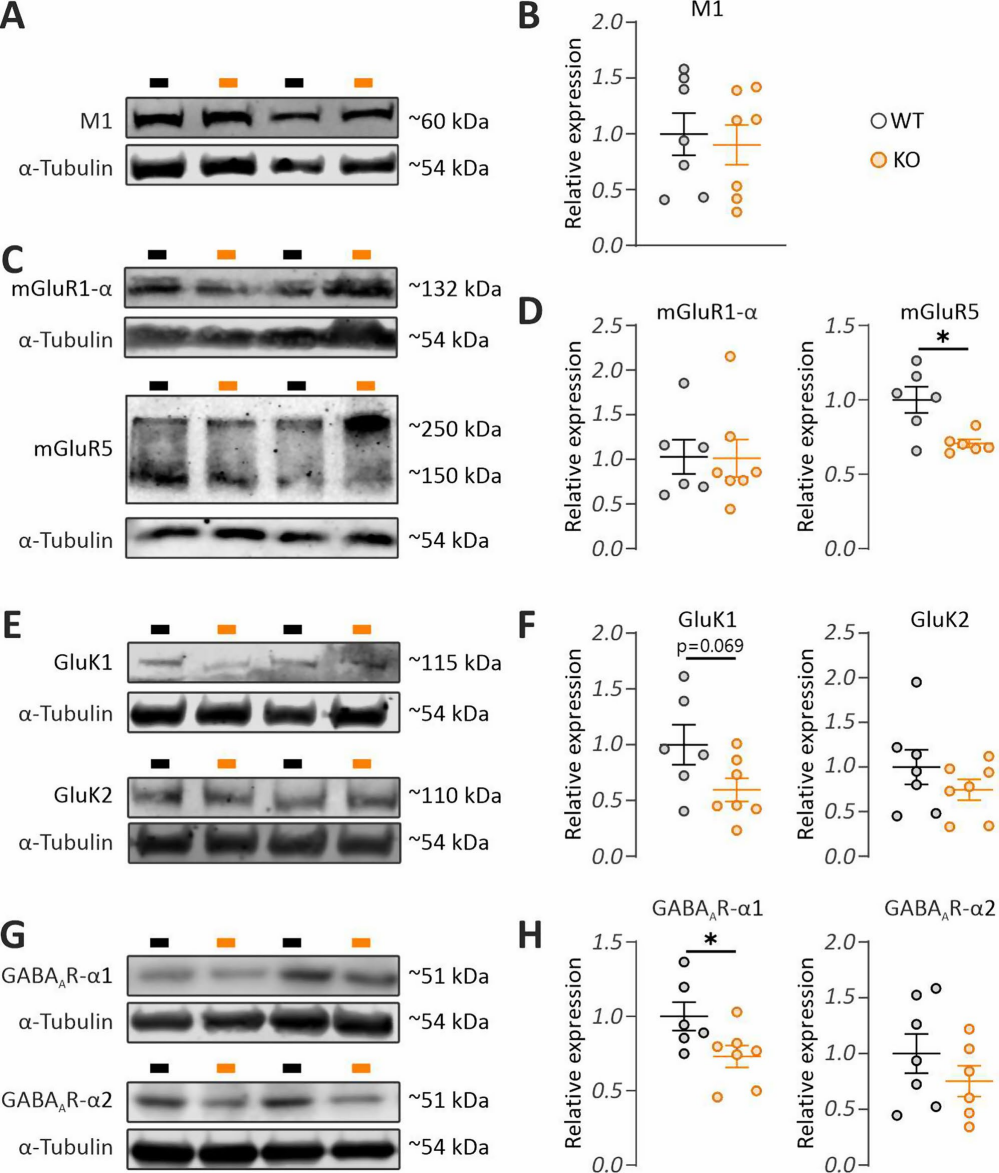
mGlur5 and GABA_A_R-α1 expression is reduced in the hippocampus of *Fmr1* KO mice. Representative western blots (**A**, **C**, **E**, **G**) and quantification graphs (B, D, F, H) are shown for: (**A**-**B**) M1 subunit of muscarinic acetylcholine receptors (mAchr), (**C**-**D**) mGlur1- α and mGlur5 (group I metabotropic glutamate receptors), (**E**-**F**) GluK1 and GluK2 subunits of kainate receptors (KAR), and (**G**-**H**) α1 and α2 subunits of GABA_A_ receptors (GABA_A_R). Notably, significant reductions were observed in mGlur5 and GABA_A_R-α1 expression, with a trend toward decreased GluK1 levels. Statistical comparisons were made using Student’s two-tailed t-test (panels **B**, **D**, **F**, **H**). Data are presented as mean ± SEM, with individual data points representing biological replicates (WT: *N* = 6–7 mice, KO: *N* = 6–7 mice). *p < 0.05

To determine whether receptors involved in the induction of distinct in vitro gamma oscillations are differentially regulated in *Fmr1* KO mice, we quantified their levels in synaptically enriched tissue (Supplementary Fig. 3) from the ventral-to-mid hippocampus corresponding to the region where gamma oscillations were recorded. We found no evidence for altered expression of the M1 mAChR, which is required for the induction of cholinergic gamma oscillations (Fig. 7A–B; T [12] = 0.3681, *p* = 0.7192). However, among Group I metabotropic glutamate receptors (mGluRs), which are activated by DHPG, mGluR5 expression was significantly reduced (Fig. 7C-D; T(10) = 3.172, p = 0.01, with a large effect size d = 1.83, 95% CI [0.44, 3.21]), while mGluR1α levels remained unchanged (Fig. 7C–D; T [11] = 0.0545, *p* = 0.9575). Analysis of KARs revealed a trend toward reduced GluK1 expression (Fig. 7E–F; T [11] = 2.016, *p* = 0.0689), though this did not reach statistical significance, and no significant change in GluK2 levels (Fig. 7E–F; T [12] = 1.116, *p* = 0.2864). Interestingly, quantification of GABA_A_R subunits, whose activation is essential for gamma oscillation induction both in vitro and in vivo, showed a significant reduction in the α1 subunit (Fig. 7G–H; T [11] = 2.266, *p* = 0.0446, with a large effect size d = 1.26, 95% CI [0.049, 2.47]), while α2 expression was unaltered (Fig. 7G–H; T [11] = 1.076, *p* = 0.3049). Overall, these findings suggest that the expression of key receptors implicated in the generation of in vitro gamma oscillations is only partially altered in *Fmr1* KO mice. The observed reductions do not fully align with the enhanced gamma oscillations seen in these animals implying that alterations in downstream signaling pathways or compensatory mechanisms aimed at dampening receptor expression may underlie the observed gamma oscillopathy.

## Discussion

In the current study, we took advantage of the well-established in vitro slice models for the analysis of gamma oscillations in a mouse model of FXS. We used three experimental protocols for the induction of gamma oscillations in vitro which involve pharmacological activation of either mAChR, mGluR or KAR (Fig. 1). Aberrant signaling mediated by activation of these receptors has been linked to the pathogenesis of FXS [6, 7, 10–12, 15]. Thus, this approach allowed us to assess the potential contribution of these signaling pathways to the generation of hypersynchronous gamma oscillations in the *Fmr1* KO mouse model. Strikingly, we found that the activation of either mAChR or mGluR leads to an increase in the power of gamma oscillations in the hippocampus of *Fmr1* KO. On the other hand, pharmacological activation of KAR leads to rather desynchronized gamma oscillations and a mild reduction in the peak power of gamma oscillations suggesting a potential loss of KAR function in the *Fmr1* KO mouse. These changes were associated with altered cellular firing patterns during gamma oscillations induced by either CCh or KA. We further identified a mild reduction in the incidence of spontaneous hippocampal SW-R events, LFP patterns which are linked to memory consolidation and appear during reduced subcortical neuromodulation [39, 67]. Of note, we found no evidence for altered baseline synaptic transmission at the CA3–CA1 synapse, and only partial changes in the expression of receptors required for the generation of gamma oscillations in vitro. Together, our data suggest that the aberrant activation of mAChR, mGluR or KAR may directly contribute to the gamma oscillopathy and associated behavioral abnormalities observed in FXS.

The most striking finding of the current study is the abnormal increase in the amplitude of cholinergic gamma oscillations in the hippocampal CA3 of *Fmr1* KO mouse (Fig. 2). Indeed, increased cholinergic tonus is associated with the emergence of LFP gamma oscillations in the hippocampus in vivo and in vitro [41, 45, 68]. However, ACh metabolism appears to be normal in male *Fmr1* KO mice suggesting that the increased gamma oscillation power observed in FXS is not due to an overall increase in the availability of ACh [69]. Of note, generation of CCh-induced gamma oscillations depends on the activation of M1-AChR [45, 49]. Accordingly, an exaggerated CCh-induced hippocampal LTD mediated by M1-AChR is evident in the *Fmr1* KO mouse [11]. Of note, we were unable to detect an increase in the expression of M1-AChR in the synaptically enriched fraction of hippocampal tissue (Fig. 7) likely because any potential increase was masked by the presence of other cell types in the whole hippocampal tissue, as demonstrated previously [6]. While proteomic studies have reported increased expression of M4 muscarinic receptors in the FXS hippocampus [6], evidence from M4 knockout mice indicates that cholinergic gamma oscillations induced by similar in vitro protocols remain normal [49], making it unlikely that M4 changes are the primary driver of the phenotype reported here. Taken together, these findings suggest that the aberrant increase in hippocampal gamma power in FXS is more likely attributable to elevated M1-AChR expression and/or abnormal activation of downstream signaling pathways.

Similar to cholinergic gamma oscillations, we found a strong enhancement of mGluR1/5 agonist DHPG-induced gamma oscillations in the hippocampal CA3 of *Fmr1* KO mice (Fig. 3). Our results conform with previous studies [50] demonstrating a higher gamma peak frequency of DHPG-induced gamma oscillations in comparison to the other pharmacologically-induced gamma oscillations. These findings also align well with the enhanced mGluR signaling and mGluR-dependent LTD reported numerous times in the hippocampal CA1 of the *Fmr1* KO mice [7–9, 70, 71]. Of note, antagonism of mGluR appears to have no effect on both CCh-induced LTD and gamma oscillations in vitro [11, 45, 50]. Furthermore, activation of either mGluR or M1-AChR leads to the enhancement of protein-synthesis dependent LTD via a common mechanism that is under the translational control of FMRP [11]. Thus, synergistic actions of aberrant mGluR and M1-AChR signaling likely contribute to the enhanced gamma oscillations in the *Fmr1* KO mice.

It is important to note that several studies have reported reduced expression of mGluR5 in *Fmr1* KO mice [72] as well as in individuals with Fragile X Syndrome [73, 74]. Building on these findings, we now demonstrate that mGluR5 protein levels are also reduced in the ventral-to-mid hippocampus. Interestingly, this reduction appears to contradict the enhanced DHPG-induced gamma oscillations observed in *Fmr1* KO mice. Therefore, the decreased mGluR5 expression in the *Fmr1* KO mice may reflect a compensatory mechanism in response to already elevated mGluR5-mediated signaling.

Notably, KA-induced gamma oscillations were differently affected in the *Fmr1* KO mice (Fig. 4), showing reduced gamma synchronization and lower peak power. Previous pharmacological work has shown that KA-induced gamma oscillations do not require activation of either mAChRs or mGluRs, suggesting that the changes we observe are unlikely to be mediated via these receptor systems [52]. While reduced synaptic expression and function of KARs has been reported in the cortex of *Fmr1* KO mice [15], direct evidence for altered KAR expression or function in the hippocampus is lacking. In our study, we detected only a non-significant trend toward reduced GluK1 expression (Fig. 7), which may reflect the predominant expression of GluK1 in hippocampal interneurons [75] and the consequent signal dilution in whole-tissue analyses. Given the absence of direct functional data, we refrain from inferring a causal role of altered KAR function in the gamma oscillation changes reported here. Instead, these alterations could be directly related to changes in GABAergic function, as suggested by the robust reduction in GABA_A_R-α1 expression observed in *Fmr1* KO hippocampus. Future studies using cell-type-specific approaches will be essential to determine the respective contributions of KARs and GABAergic signaling to these network alterations.

CA3 LFP gamma oscillations represent compound synaptic potentials recorded extracellularly and their generation requires activity of both pyramidal cells and GABAergic interneurons [53, 54]. Theoretical and experimental work indicate that distinct in vitro network oscillations require different levels of inhibitory and excitatory fast synaptic transmission. Blockade of GABA_A_R-mediated fast inhibitory transmission eliminates gamma oscillatory activity in all in vitro models measured in the current study [45, 50, 52]. Drugs that prolong the fast inhibitory synaptic currents, such as zolpidem and pentobarbital, reduce the gamma peak frequency of KA- and DHPG-induced gamma oscillations [50, 52], whereas this frequency shift is not observed for CCh-induced gamma oscillations [50]. On the other hand, blockade of AMPAR-mediated fast excitatory synaptic transmission leads to complete elimination of CCh- and DHPG- induced gamma oscillations [45, 50] without any major effect on KA-induced gamma oscillations [52]. Collectively, with regards to the mouse model of FXS, these findings have several implications [1]: in vitro gamma oscillations (CCh- and DHPG-induced) which depend on an intact AMPAR-driven excitatory synaptic transmission are elevated [2], inhibition-driven KA-induced gamma oscillations are reduced in their synchrony and show an increase in gamma peak frequency [3], excitation-inhibition balance during gamma oscillations may underlie the distinct changes in the properties of gamma network activity induced by CCh, DHPG or KA in the hippocampal CA3 of *Fmr1* KO mice. Accordingly, hippocampal CA3 neurons of the *Fmr1* KO mice appear to be hyperexcitable and gamma-range stimulation of CA3 fibers leads to an aberrant augmentation of the excitatory neurotransmission at the CA3–CA1 synapses [76, 77]. Several studies also provide evidence for an altered excitation-inhibition balance in the *Fmr1* KO mice [78–80]. Consistent with these findings, we now report reduced protein expression of the α1-subunit-containing GABA_A_ receptors, which mediate fast phasic inhibition (Fig. 7). Therefore, future studies investigating alterations in excitation-inhibition balance during ongoing network oscillations at the single-cell level-using approaches such as patch-clamp electrophysiology may offer deeper insights into the underlying mechanisms.

Parvalbumin-positive (PV+) inhibitory interneurons are indispensable for generation and maintenance of gamma oscillations both in vivo and in vitro [53, 54]. PV+ interneuron dysfunction and reduction in their number have been reported in the *Fmr1* KO mice [21, 81–83] and in postmortem neocortical tissue of autistic individuals [84]. Paradoxically, deficient PV+ neuron function can lead to increased broadband gamma power, however, with a desynchronized cellular activity to the ongoing LFP oscillations [85]. Indeed, despite the overall increase in the pyramidal cell firing [25], such desynchronization of cellular activity within the CA1 network and to the ongoing LFP oscillations have been demonstrated in the *Fmr1* KO mice [24, 86]. In line with these findings, our analysis of extracellular unit firing in a gamma-cycle dependent manner revealed an aberrant cellular firing pattern during gamma oscillations induced by CCh or KA. We identified an aberrant increase in unit firing, most probably representing the action potentials generated by principal pyramidal cells, during the late stages of gamma cycles induced by CCh. On the other hand, a reduction in cellular firing was evident, when gamma oscillations were induced by KA. However, it should be noted that while most of the detected units in the pyramidal layer likely originate from pyramidal cells, contributions from nearby interneurons cannot be excluded. Focusing on the upper quartile of spike amplitudes likely emphasizes units closer to the electrode, reducing—but not eliminating—signal overlap. Nevertheless, increased CCh-induced gamma oscillations might reflect an aberrant increase in cellular firing leading to an enhanced broadband gamma power as observed in vivo [24, 85, 86]. In addition, higher spike amplitudes in *Fmr1* KO slices under CCh could indicate increased neuronal recruitment or synchrony, potentially due to reduced inhibitory tone or altered intrinsic excitability previously reported in *Fmr1* KO mice [87, 88]. On the other hand, reduced synchronization of gamma oscillations induced by KA might be associated with the desynchronization of underlying cellular activities (reduced unit firing) together with a potentially reduced KAR function in the hippocampus which was not directly assessed in this study.

Interestingly, we could not identify such an altered unit firing during DHPG-induced gamma oscillations. The increase in gamma power observed during DHPG-induced gamma oscillations in the *Fmr1* KO mice was rather small in comparison to CCh-induced gamma oscillations. In addition, our analysis of extracellular unit firing might not detect changes in subcellular activities (e.g. synaptic potentials or “spikelets”) which can also influence gamma oscillations strength. Hence, future studies combining single cell and extracellular electrophysiology will help resolving cellular correlates of increased DHPG-induced gamma oscillations in the *Fmr1* KO mice.

Our results are in line with recent in vivo studies showing that *Fmr1* KO mice exhibit a pronounced dominance of CA3-driven slow gamma oscillations (20–50 Hz) in the CA1 region [24–27]. Consistently, our in vitro recordings from the CA3 region demonstrate a robust increase in gamma oscillations induced by both CCh and DHPG, with peak frequencies within the same 20–50 Hz range (Figs. 2 and 3). Importantly, this predominance of CA3-driven slow gamma oscillations has been associated with impaired memory encoding and retrieval, ultimately leading to deficits in cognitive flexibility [26, 27, 86]. These converging in vivo and in vitro findings highlight that gamma oscillations measured in our slice experiments might serve as reliable, behaviorally relevant physiological read-outs, providing a meaningful link between network-level alterations and cognitive outcomes in *Fmr1* KO mice.

We focused on 4–5-week-old mice to align our in vitro slice experiments with the age range most frequently used in in vitro electrophysiological studies investigating *Fmr1* KO network function [89–91]. Considering the persistent of hippocampal gamma oscillopathies reported in in vivo studies of adult *Fmr1* KO mice [24–27], the findings we report here likely reflect enduring circuit dysfunction rather than transient developmental phenomena. Future work should systematically test whether these oscillatory alterations remain stable across the lifespan.

Under low cholinergic tonus, for example during slow wave sleep, hippocampal LFP activity is dominated by SW-R [92, 93]. These LFP oscillations have been linked to the replay of memory-related cellular activity, thus, supporting memory consolidation [94, 95]. In slice preparations including the transverse sections of ventral-to-mid portion of the hippocampus, spontaneous SW-R activity also emerges and can be used as proxy to those that occur in vivo [40, 42, 43]. We found an overall reduction in the incidence of hippocampal SW-R in vitro (Fig. 5). As aforementioned for gamma oscillations, generation and incidence of SW-R activity depends on functionally-intact PV+ interneurons [38, 96]. Thus, our results fit well with the potentially reduced PV+ interneuron function in the *Fmr1* KO mice leading to reduced SW-R incidence.

To the best of our knowledge, only one study investigated hippocampal SW-R in the *Fmr1* KO mice demonstrating a reduced SW-R duration and ripple frequency without any alteration in SW incidence in the dorsal CA1 in vivo [25]. Importantly, another in vitro study using an *Fmr1* KO rat model, shows convincing evidence for a specific reduction in SW incidence in the dorsal CA1 but not in the ventral CA1 [97]. Our slice preparation also included slices from the mid portion of the hippocampus which might explain the slight reduction we observed in SWR incidence in our data set. Taken together, we propose that discrepancies across the previous and current studies may stem from differences in experimental conditions (in vivo vs. in vitro), hippocampal segment (dorsal vs. ventral), and subregions examined (CA3 vs. CA1) and merits further investigation.

Interestingly, we found no evidence for altered baseline synaptic excitability, as measured by fEPSP slopes at the CA3–CA1 synapse, consistent with previous findings in an *Fmr1* KO rat model [97]. This suggests that the altered hippocampal network oscillations observed in *Fmr1* KO mice are not attributable to changes in basal synaptic transmission at the CA3–CA1 synapse. Importantly, *Fmr1* KO slices exhibited elevated baseline gamma power compared to WT, although this baseline was much lower than pharmacologically induced oscillations. This indicates that the robust genotype differences observed during pharmacologically induced gamma oscillations cannot be explained by baseline activity alone, but instead reflect enhanced responsiveness of gamma-inducing signaling pathways. These findings further suggest that glutamatergic metabotropic and cholinergic signaling may already be more engaged under resting conditions in *Fmr1* KO slices, predisposing the network to generate stronger oscillatory activity upon stimulation.

## Limitations and conclusions

Taken together, the current study demonstrates that activation of either mAChR or mGluR is sufficient to mimic the aberrant increase in the gamma oscillations observed in the hippocampus of the *Fmr1* KO mice in vivo. It should be noted that the increase in gamma oscillation power has also been observed in numerous cortical areas including the auditory, somatosensory, frontal and temporal cortex of the *Fmr1* KO mice [18–23]. Whether these oscillatory alterations can be mimicked using pharmacologically-induced gamma oscillations in cortical slice preparations [98] needs to be investigated in future. This will warrant the robust testing of potential pharmaceuticals in the treatment of neuronal hyperexcitability in FXS. It is important to note that human EEG studies have reported a similar enhancement of gamma power in individuals with FXS [28–30, 99, 100]. Thus, utilization of gamma oscillation power as a mesoscopic biomarker may pave the way in the diagnosis of neurodevelopmental disorders such as FXS and facilitate target identification for the treatment of neuronal hyperexcitability in FXS. Our finding of altered multi-unit activity, including differences in activation patterns, did not allow differentiation between inhibitory interneurons and excitatory pyramidal cells. Future studies might employ single-cell or tetrode recordings to further investigate the underlying cell-type–specific activation patterns.

## Electronic supplementary material

Below is the link to the electronic supplementary material.

Supplementary material 1

## Data Availability

The datasets used and/or analyzed in the current study are available from the corresponding author on reasonable request.

## Abbreviations

aCSF: artificial Cerebrospinal Fluid
CA: Cornu Ammonis
CCh: Carbachol
DHPG: Dihydroxyphenylglycine
EEG: Electroencephalogram
fEPSP: field Excitatory Postsynaptic Potential
FMRP: Fragile X mental retardation protein
Fmr1 KO: Fragile X mental retardation gene 1 knockout
FXS: Fragile X syndrome
I-O: Input-Output
KA(R): Kainate (Receptor)
LFP: Local Field Potential
LTD: Long-Term Depression
mGluR: metabotropic Glutamate Receptor
mAChR: muscarinic Acetylcholine Receptor
PP: Paired-Pulse
SC: Schaffer Collaterals
SP: Stratum Pyramidale
SW-R: Sharp Wave-Ripples
WT: Wild-Type
