# Supplementary material for: Aberrant hippocampal gamma oscillations in a mouse model of fragile X syndrome: insights from in vitro slice models
Source: Mol Autism. 2025 Nov 3;16:55. doi: 10.1186/s13229-025-00687-9 (PMC12581432; doi:10.1186/s13229-025-00687-9)
Supplement: Supplementary file 1 — Supplementary material 1 [file 13229_2025_687_MOESM1_ESM.pdf]

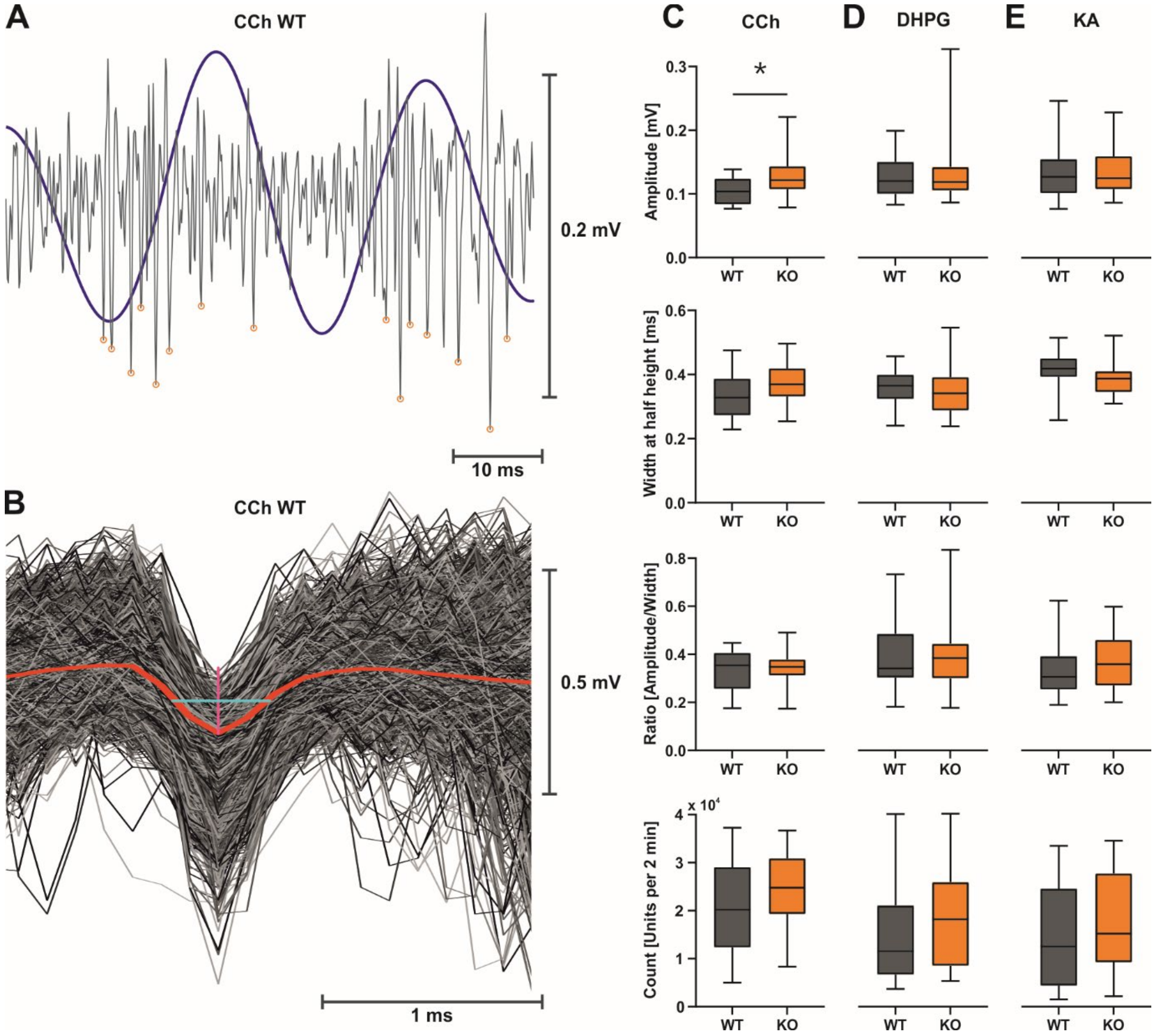

Supplementary Figure 1 (Supplement to Main Figure 2-4): Details of multi-unit activity (MUA) extraction and statistics of key features of individual units. (A) Sample trace of gamma oscillations induced via bath-application of Carbachol (CCh, 5 µM) illustrating multi-unit extraction; blue: 15-45 Hz component (FFT band-pass filter) representing gamma rhythm, black: 500-3000 Hz component (FFT band-pass filter) representing MUA, orange circles: spikes marked for further extraction and analysis. **(B)** All detected spikes (as shown in **A**) were isolated (shades of grey) to determine amplitude (magenta) and width at half height (light blue). Please note, the median (red) is only shown for reference and illustration of how amplitude and width were calculated. All parameters were calculated for individual units, medians were then joined and used for statistical evaluation of multi-unit activity in datasets as shown in **C-E**. **(C)** Features of MUA during gamma oscillations induced via CCh in WT and KO (WT: n = 12 / N = 6, KO: n = 25 / N = 8). While all other parameters were unchanged, the amplitude of units was significantly higher in KO compared to WT. **(D)** Features of MUA during gamma oscillations induced via DHPG in WT and KO (WT: n = 20 / N = 8, KO: n = 24 / N = 8). MUA was comparable among WT and KO. (**E**). Features of MUA during gamma oscillations induced via KA in WT and KO (WT: n = 27 / N = 6, KO: n = 25 / N = 6). MUA was comparable among WT and KO. Statistical comparison was performed using Student´s two-tailed t-test in: **C** for Width (T(33) = 1.710, p = 0.0967), Ratio (T(33) = 0.3762, p = 07091), and Count (T(33) = 1.325, p = 0.1944) in **D** and **E** for Width (DHPG: T(42) = 0.2402, p = 0.8114; KA: T(50) = 1.621, p = 0.1113) and Mann-Whitney U test in: **C** for Amplitude (p = 0.0454), in **D** and **E** for Amplitude (DHPG: p = 0.9907; KA: p = 0.7853), Ratio (DHPG: p = 0.6323; KA: p = 0.2664), and Count (DHPG: p = 0.1485; KA: p = 0.2166). Data are presented as boxplots, where the central line indicates the median, the box spans the interquartile range (IQR; 25th–75th percentile), and whiskers extend to the highest and lowest datapoints. *p <0.05.

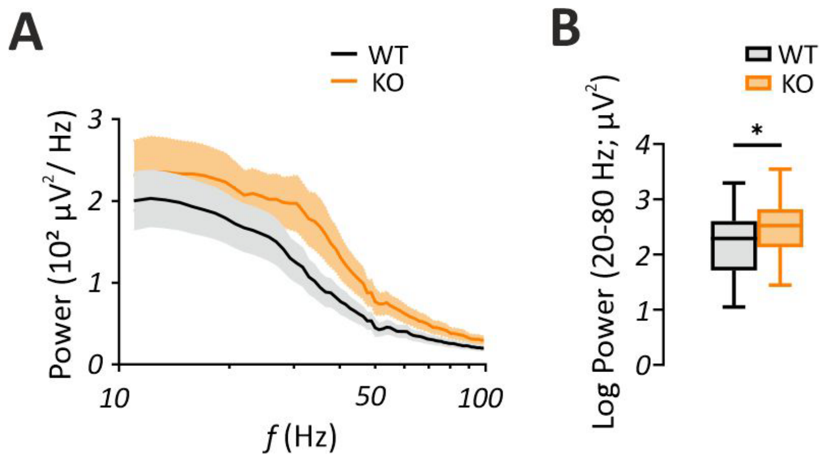

Supplementary Figure 2 (Supplement to Main Figure 2-5): Baseline gamma power (20-80 Hz) is increased in the hippocampal CA3 region of *Fmr1* KO mice. (A) Average power spectrum showing a mild increase in the power of gamma-range frequencies. Note that the *f* is shown in logarithmic scale. **(B)** Summary plot illustrating a significant increase in the overall power (20-80 Hz) (WT: n = 43 / N = 11, KO: n = 54/ N = 14). Statistical comparison was performed using Student´s two-tailed t-test in **B**. Data are presented in **A** as mean ± SEM and in **B** as Tukey-style boxplots, where the central line indicates the median, the box spans the interquartile range (IQR; 25th–75th percentile), and whiskers extend to 1.5 x IQR. *p <0.05.

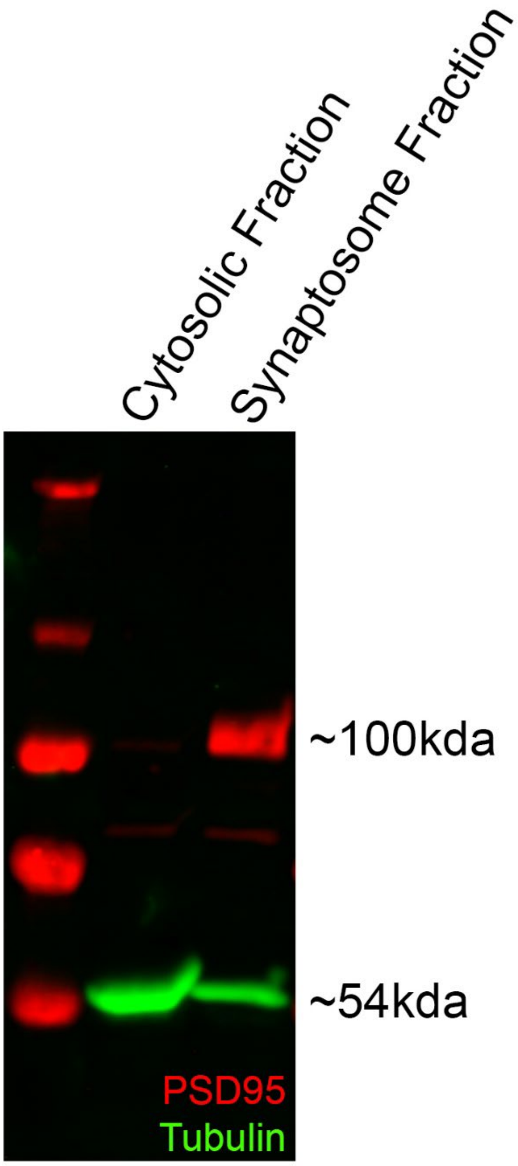

Supplementary Figure 3 (Supplement to Main Figure 7): Western blot analysis of fractions obtained from the synaptic enrichment protocol demonstrates a clear enrichment of the synaptic protein PSD95 in the synaptosomal fraction.
